# Case of Asphyxia Produced by Compression of the Navel-String during Birth, Removed by Insufflation of the Lungs

**Published:** 1820-06

**Authors:** 

**Affiliations:** Chalons-sur-Saone.


FOR THE LONDON MEDICAL AND PHYSICAL JOURNAL.

### *Case of Asphyxia produced by Compression of the Navel-string during Birth, removed by Insufflation of the Lungs.* By Dr. PERNET, of Chalons-sur-Saone. Communicated to the Society of the Faculty of Medicine of Paris.

ON the 15th of March, 1817, I was called, at two o'clock in the morning, to visit a woman, who was seven months and a half gone with child, and who was said to have a prolapsus of the rectum, produced by efforts made on evacuating the intestines.

On my arrival, I found, instead of the pretended prolapsus of the rectum, the lower extremities and part of the trunk of a fetus protruding from the vagina, and which had been in that situation since half-past ten o'clock in the evening, since when the patient had been lying on the bed. The delivery was immediately completed.

The asphyxia of the infant appeared to be complete. There was no sensible motion of the heart. The body was almost cold, and the limbs remained in any position in which they were placed. I at first abandoned it, to proceed to the extraction of the placenta, which adhered strongly to the uterus; after which I directed my attention to the child, which had been put aside as dead by the attendants, after they had employed the usual restorative means.

Having no better tube at hand, I employed a quill, with which the lungs were inflated, in the way of artificial respiration; and this process was continued for a quarter-of-an-hour without success. I then made new attempts to remove the placenta; but, it being still firmly retained, I determined to wait yet longer, and recommenced the same operation on the infant, which was pursued for a quarter of an hour longer without success. It was now interrupted whilst I turned to the mother to remove the placenta, which became detached from the uterus.

Artificial respiration was again established in the infant, and continued for half an hour, when, just as I had abandoned it, having lost all hope of its success, a short and very slight respiratory effort took place at the instant after I had ceased my insufflations. They were consequently renewed, and voluntary inspirations were effected as soon as I ceased to practise it. They were repeated only every five minutes; so that I was obliged to persevere in the use of the same means during an hour longer. At length, the voluntary inspirations succeeded each other more rapidly; respiration was established, although in a very short and laborious manner. The infant was placed in a warm bath: the state of asphyxia returned, but some new insufflations completely removed it. The infant was now carried into a very warm room, given the next day to a good nurse, and now enjoys the most perfect health.

